# Hydroxyapatite microbeads containing BMP-2 and quercetin fabricated via electrostatic spraying to encourage bone regeneration

**DOI:** 10.1186/s12938-023-01078-y

**Published:** 2023-02-18

**Authors:** Seoyun Lee, Honghyun Park, Jeong-Seop Oh, Kyubin Byun, Dae-Yong Kim, Hui-suk Yun, Byung-Jae Kang

**Affiliations:** 1 Department of Veterinary Clinical Sciences, College of Veterinary Medicine and Research Institute for Veterinary Science, Seoul National University, Seoul, 08826 South Korea; 2 BK21 FOUR Future Veterinary Medicine Leading Education and Research Center, Seoul National University, Seoul, 08826 South Korea; 3 Department of Advanced Biomaterials Research, Ceramics Materials Division, Korea Institute of Materials Science (KIMS), Changwon, 51508 South Korea; 4 Department of Veterinary Pathology, College of Veterinary Medicine and Research Institute for Veterinary Science, Seoul National University, Seoul, 08826 South Korea; 5 Department of Advanced Materials Engineering, University of Science & Technology (UST), Daejeon, 34113 South Korea

**Keywords:** Hydroxyapatite microbeads, Electrostatic spraying, Bone regeneration, Bone morphogenetic protein-2, Quercetin

## Abstract

**Background:**

Hydroxyapatite (HAp) possesses osteoconductive properties, and its granular form can serve as an effective drug delivery vehicle for bone regeneration. Quercetin (Qct), a plant-derived bioflavonoid, is known to promote bone regeneration; however, its comparative and synergistic effects with the commonly used bone morphogenetic protein-2 (BMP-2) have not been investigated.

**Methods:**

We examined the characteristics of newly formed HAp microbeads using an electrostatic spraying method and analyzed the in vitro release pattern and osteogenic potential of ceramic granules containing Qct, BMP-2, and both. In addition, HAp microbeads were transplanted into a rat critical-sized calvarial defect and the osteogenic capacity was assessed in vivo.

**Results:**

The manufactured beads had a microscale size of less than 200 μm, a narrow size distribution, and a rough surface. The alkaline phosphatase (ALP) activity of osteoblast-like cells cultured with the BMP-2-and-Qct-loaded HAp was significantly higher than that of either Qct- or BMP-2-loaded HAp groups. The mRNA levels of osteogenic marker genes such as ALP and runt-related transcription factor 2 were found to be upregulated in the HAp/BMP-2/Qct group compared to the other groups. In micro-computed tomographic analysis, the amount of newly formed bone and bone surface area within the defect was significantly higher in the HAp/BMP-2/Qct group, followed by the HAp/BMP-2 and HAp/Qct groups, which is consistent with the histomorphometrical results.

**Conclusions:**

These results imply that electrostatic spraying can be an efficient strategy to produce homogenous ceramic granules and that the BMP-2-and-Qct-loaded HAp microbeads can serve as effective implants for bone defect healing.

**Supplementary Information:**

The online version contains supplementary material available at 10.1186/s12938-023-01078-y.

## Background

Large bone defects constitute a serious clinical concern and can occur as a result of trauma, cancer, or congenital diseases. To repair large bone defects in orthopedic and dental surgery, various types of bone grafts are used in clinical practice, such as autografts, allografts, and synthetic bone grafts. Among them, autografts have been considered as standard treatment; however, they are known to suffer from many complications, such as donor site infection and inflammation, limited yield, and persistent discomfort [1]. In addition, allografts are associated with minor immunogenic rejection and the possibility of disease transmission [2]. Therefore, synthetic bone grafts that can stimulate bone regeneration and replace current treatments have become necessary.

In this regard, various materials have been studied as synthetic bone grafts, including calcium phosphates, calcium sulfates, bioactive glass, polymers, and metals [3–7]. The optimal bone substitute should be able to provide an osteoconductive scaffold and osteoinductive growth factors, and should be structurally similar to natural bone [8]. Among these candidates, hydroxyapatite (HAp) is the most widely used synthetic form of calcium phosphate, a major inorganic component of bone, owing to its biocompatibility, osteoconductivity, and adsorption properties [9]. In addition, HAp is known to cause no cytotoxicity, no inflammatory response, and promotes cell adhesion and growth when clinically applied [10]. Given its rapid absorption and large surface area, granular application of HAp is the most advantageous for drug encapsulation among the various forms of HAp application [11].

HAp granules can be manufactured in various ways, including spray drying [12] and emulsion techniques [13]. However, the numerous settings and instability of heat-labile drugs during the drying process make it difficult for the conventional spray-drying method to maintain ideal conditions for granule production [14]. Furthermore, the emulsion technique typically requires a post-complex treatment to eliminate residual oil after production [15]. To address these disadvantages of existing approaches, in this study, we produced HAp microbeads via electrostatic spraying, which can be employed with simple parameters, such as nozzle size, frequency, electrode, and pressure. Although it is difficult to uniformly disperse the ceramic, two-step mixing pretreatment allowed for the production of homogenous and high-yielding HAp particles.

Bioactive growth factors must be incorporated into HAp microbeads, because synthetic bone grafts do not induce considerable bone regeneration compared with autografts [16]. Bone morphogenetic protein 2 (BMP-2), a member of the TGF family, is a potent osteogenic inducer that promotes osteogenic differentiation of osteoblast progenitors or stem cells [17]. In humans, BMP-2 exists at very low concentrations of 2 ng per 1 g of bone and a serum concentration of 90 pg/ml [18]. Unfortunately, when injected directly into a defect site, BMP-2 has a short half-life of 7–16 min, because it is rapidly degraded by proteases in vivo. Thus, a supraphysiological dose of these proteins is usually necessary to facilitate bone repair in clinical situations. However, overdosage of BMP-2 can cause undesirable side effects, such as inflammation, bone cyst formation, and osteoclast activation [19].

Owing to the aforementioned limitations, sustained release through a delivery carrier is essential for effective BMP-2 application. In addition, various growth factors and medications, such as vascular endothelial growth factor [20], bisphosphonates [21], and anti-inflammatory drugs [22], have been examined for their synergistic effects in combination with BMP-2 to reduce side effects and enhance bone regeneration. Among these factors, quercetin, a flavonoid found in fruits and vegetables, is widely used for its anti-inflammatory, anticancer, neuroprotective, and bone-regeneration properties [23–26]. Recent research has shown that quercetin enhances osteogenesis and angiogenesis while inhibiting osteoclastogenesis [27]. Since sufficient research has not been conducted on the synergistic bone-healing effect of quercetin on BMP-2, in vitro and in vivo applications of both BMP-2 and quercetin through sustained-release carriers are required.

In this study, we first developed HAp microbeads using electrostatic spraying and assessed the physicochemical characteristics of the fabricated microbeads. Second, we examined whether HAp beads containing BMP-2 and/or quercetin can function as a delivery vehicle for growth factors and can induce effective bone regeneration by evaluating the in vitro release profiles and osteoblastic differentiation activity. Finally, BMP-2 or quercetin-loaded beads were applied to the rat calvarial bone defect model to compare in vivo osteogenesis and assess the synergistic effect of both drugs in combination.

## Results

### Physical properties and surface study of fabricated HAp beads

**Fig. 1:**
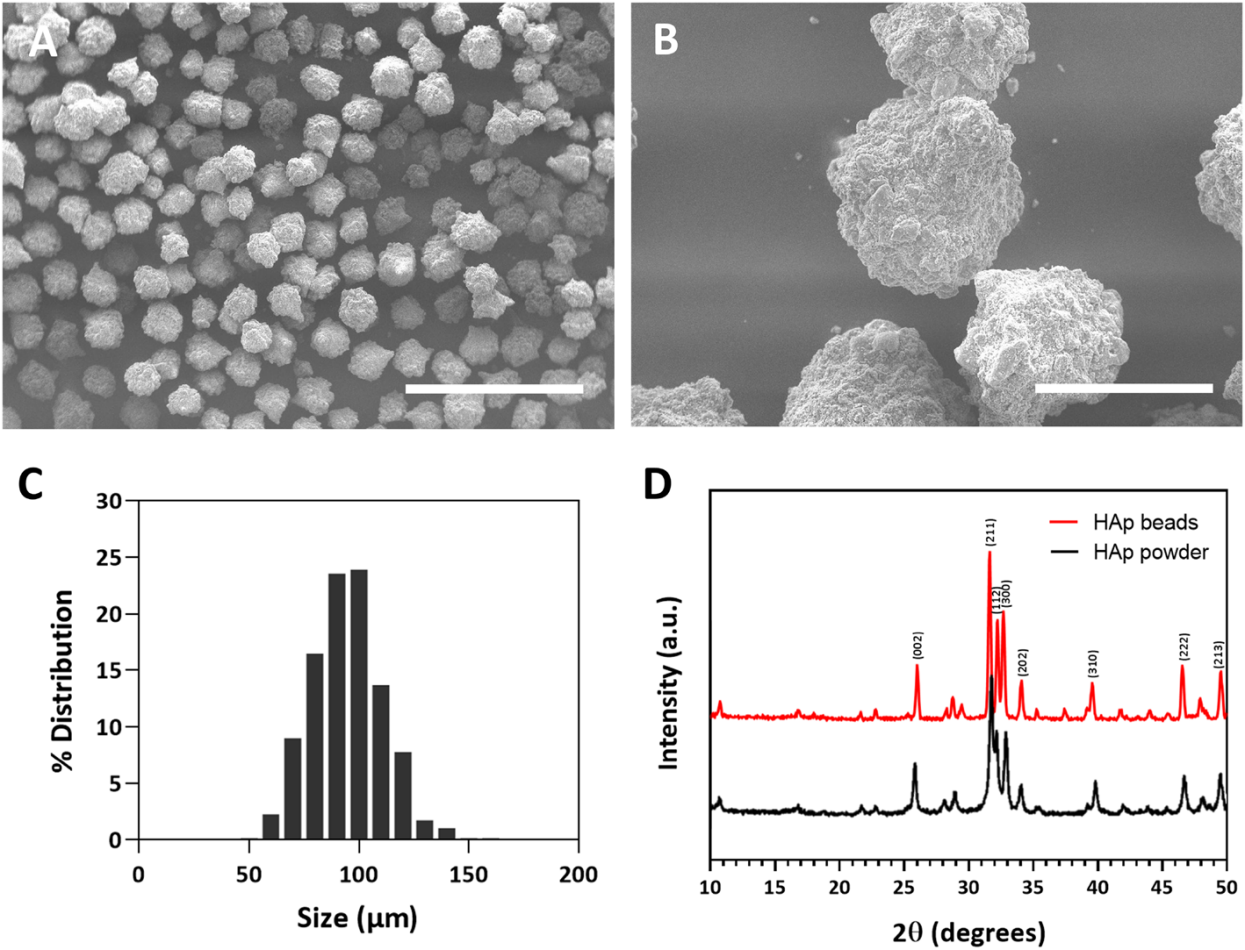
SEM images and size distribution graph of fabricated hydroxyapatite microbeads. **A,** **B** SEM images showing spherical microsized hydroxyapatite beads. A rough surface was produced on the microbeads. Scale bar = 500 μm (×100 image) and 100 μm (×500 image). **C** Size of microspheres was measured at 89.6 ± 16.1 μm, and the size distribution of the microbeads was relatively narrow. **D** XRD data showed hydroxyapatite was successfully sintered by heat treatment, which had high crystallinity

SEM images were used to investigate the shape, surface conditions, and size of the sintered hydroxyapatite microbeads. The SEM images showed the spherical shape of the microsized hydroxyapatite beads (Fig. 1A). The surface of the microbeads was rough, which was formed by agglomeration after 1200 ℃ heat treatment (Fig. 1B). The size of the microspheres was 89.6 ± 16.1 μm, which was analyzed using the SEM images via ImageJ (*n* = 577). The size distribution of the microbeads was relatively narrow, with over 78% of the beads having an average diameter of 20 μm (Fig. 1C). The XRD data showed that the crystallinity of the hydroxyapatite microbead was increased after 1200 ℃ heat treatment (Fig. 1D).

### Loading efficiency and release profiles of quercetin-and-BMP-2-containing HAp beads

**Fig. 2:**
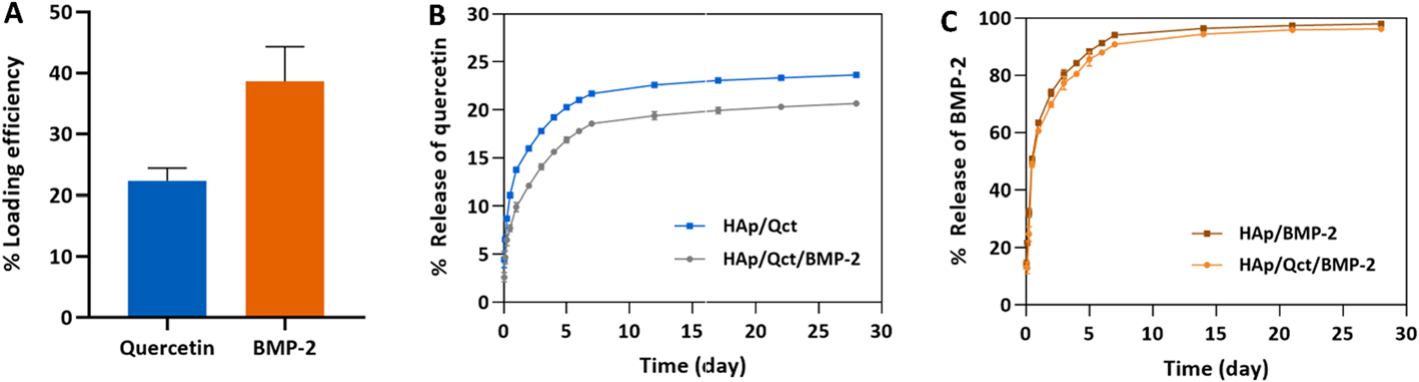
Loading efficiency and in vitro release pattern of quercetin and BMP-2 from hydroxyapatite microbeads. **A** Loading efficiency of quercetin and BMP-2 is represented. **B** Release quantity of quercetin from microbeads loaded with both quercetin and BMP-2 (gray line) and quercetin from microbeads loaded with only quercetin (blue line). **C** BMP-2 release behavior of the quercetin- and BMP-2-loaded group (orange line) and the only-BMP-2-loaded group (brown line) is shown

Loading efficiency and release behavior were investigated after loading quercetin and BMP-2 onto the microbeads (Fig. 2). To remove excess quercetin and BMP-2 without adsorption, centrifugation and PBS washing were carried out, and the loading efficiency was compared to the initial amount of loading. The loading efficiencies of quercetin and BMP-2 were 22.3 ± 2.1% and 38.6 ± 5.7%, respectively (Fig. 2A). Quercetin release behavior was evaluated with and without consecutive BMP-2 loading. The loaded quercetin was steadily released from the microbeads over 28 days. The release amount of quercetin from the quercetin-and-BMP-2-loaded microbeads was slightly lower than that of quercetin from the only-quercetin-loaded microbeads, and it was cumulatively released 20.7 ± 0.3% and 23.6 ± 0.1% over 28 days, respectively (Fig. 2B). BMP-2 release behavior was also verified for 28 days, wherein it was sustainably released over the entire period. Unlike the quercetin release behavior from the microbeads, BMP-2 release behavior was not significantly difference between the quercetin-and-BMP-2-loaded group and only-BMP-2-loaded group was not significantly different, irrespective of quercetin loading. The cumulative release of BMP-2 from the quercetin-and-BMP-2-loaded group and only-BMP-2-loaded group was 97.9 ± 0.5% and 96.2 ± 0.2%, respectively (Fig. 2C).

### Culture of MG-63 cells with HAp beads

**Fig. 3:**
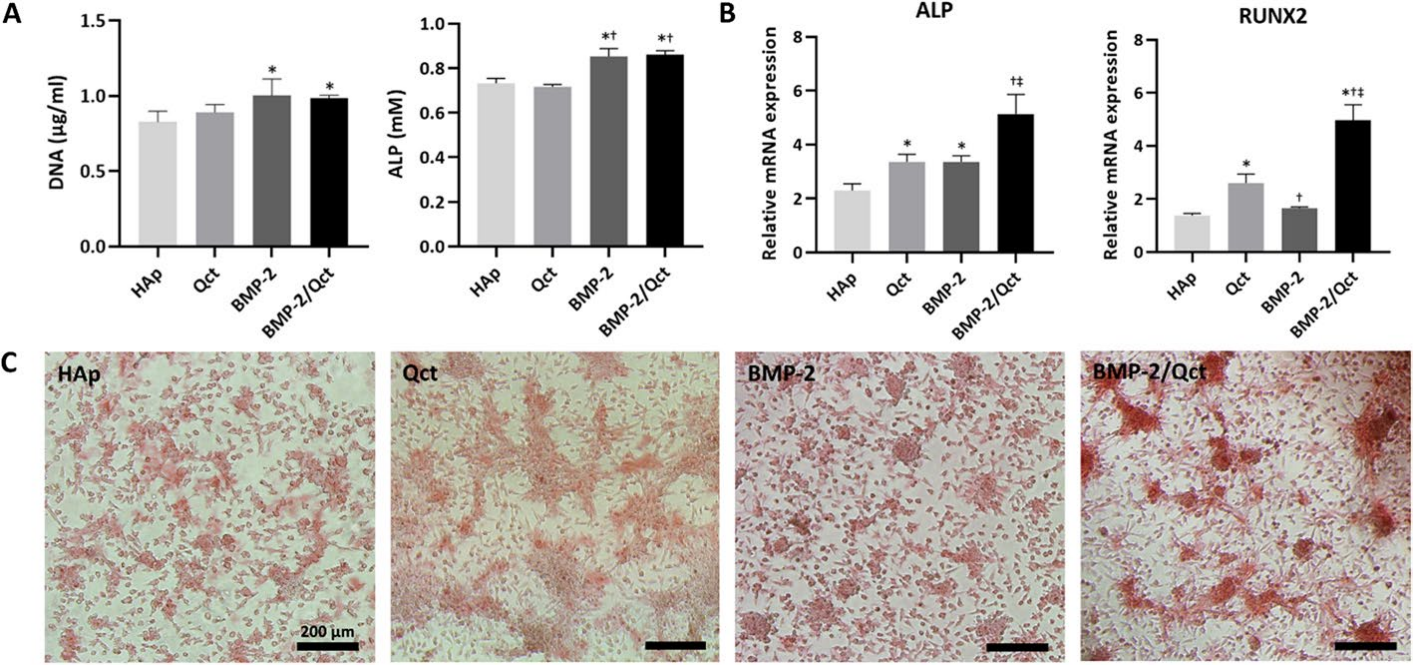
Alkaline phosphatase (ALP) activity, qRT-PCR analysis of ALP and RUNX2, and alizarin red S stain. **A** Quantification of DNA and ALP following 2 weeks of cultivation in osteogenic differentiation media. **B** Relative mRNA expression of osteogenic genes in each group. **C** Alizarin red staining reveals the production of calcium during osteogenic differentiation. *p *< 0.05 compared to the HAp (*), Qct (†), and BMP-2 (‡) groups

After 2 weeks of cultivation in osteogenic differentiation media, DNA and ALP were quantified (Fig. 3A). The DNA of BMP-2-loaded (1.0 ± 0.1 μg/ml) and the BMP-2-and-quercetin-loaded group (1.0 ± 0.1 μg/ml) was higher than that in the non-loaded group (0.8 ± 0.1 μg/ml). The DNA of the quercetin-loaded group (0.9 ± 0.1 μg/ml) was slightly higher than that of the non-loaded group; however, the difference was not statistically significant. ALP of BMP-2-loaded (0.85 ± 0.04 mM) and the BMP-2-and quercetin-loaded group (0.86 ± 0.02 mM) was significantly higher than that of the non-loaded (0.73 ± 0.02 mM) and quercetin-loaded groups (0.72 ± 0.01 mM). However, the results for the osteogenic marker gene expression were considerably different. ALP gene expression in the quercetin-loaded (3.4 ± 0.3 fold) and BMP-2-loaded groups (3.4 ± 0.2 fold) was higher than that in the non-loaded group (2.3 ± 0.2 fold). The expression of the BMP-2-and-quercetin-loaded group (5.1 ± 0.7 fold) was significantly higher than that of the other groups. RUNX2 gene expression of the quercetin-loaded group (2.6 ± 0.3 fold) was higher than that of the non-loaded (1.4 ± 0.1 fold) and BMP-2-loaded groups (1.7 ± 0.0 fold). The expression in the BMP-2-loaded group was higher than that in the non-loaded group (*p* < 0.05). Similar to ALP gene expression, RUNX2 gene expression in the BMP-2-and-quercetin-loaded group (5.0 ± 0.6 fold) was significantly higher than that in the other groups (Fig. 3B). Alizarin red staining revealed calcium formation during osteogenic differentiation. As a result of previous quantitative analyses, the BMP-2-and-quercetin-loaded group showed dramatically stronger positive signals by induction of osteogenic differentiation compared to the other groups (Fig. 3C).

## Micro-CT analysis of newly formed bone

**Fig. 4:**
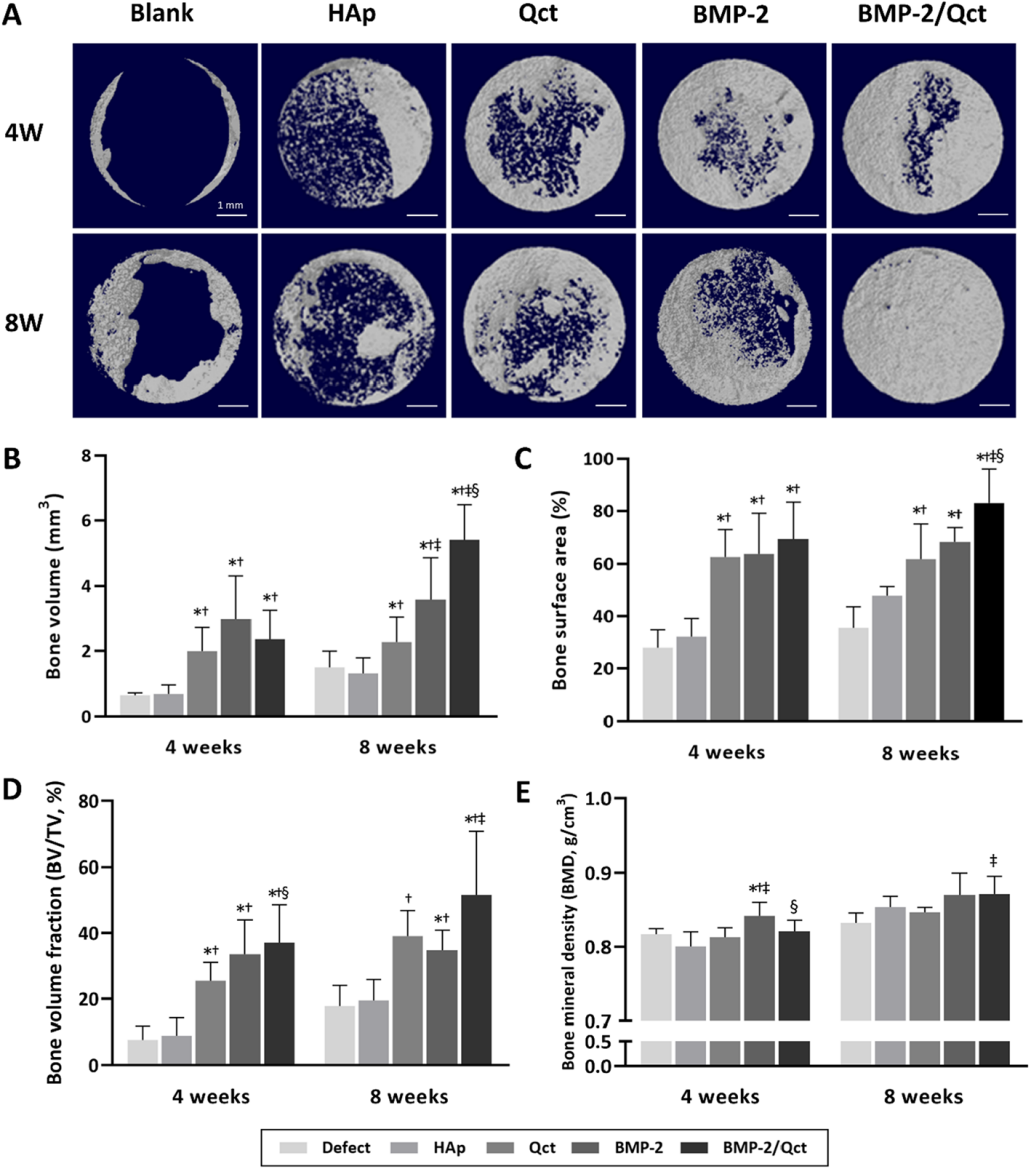
Micro-CT analysis of bone formation in rat calvarial defects. **A** 3D-reconstructed images of each group at 4 and 8 weeks. Quantitative analysis of **B** bone volume (BV), **C** bone surface area, **D** bone volume fraction (BV/TV), and (**E**) bone mineral density (BMD) within defects. Scale bar = 1 mm. *p* < 0.05 compared to the blank (*), HAp (†), Qct (‡), and BMP-2 (§) groups

As shown in the 3D reconstructed images, the control group showed little bone growth along the margin of the host bone, and no bone bridging was observed, indicating that the defect was critically sized (Fig. 4A). At 4 weeks following transplantation, the BMP-2 group showed higher bone volume than in the other groups. In contrast, the BMP-2/Qct group (5.40 ± 1.09 mm^3^) had significantly greater new bone formation than all other groups at 8 weeks, followed by the BMP-2 (3.58 ± 1.30 mm^3^) and quercetin groups (2.27 ± 0.76 mm^3^), respectively (Fig. 4B). Furthermore, the bone surface area, which indicates the percentage of bone growth area within the defect area, and bone volume fraction were the highest in the BMP-2/Qct group at both 4- and 8-week post-implantation (Fig. 4C, and D). Bone mineral density was considerably higher in the BMP-2 group at week 4 than in the other groups; at week 8, both the BMP-2/Qct (0.87 ± 0.02 gHA/cm^3^) and the BMP-2 groups (0.87 ± 0.03 gHA/cm^3^) exhibited comparable tendencies, but only the simultaneously applied group demonstrated significance relative to the Qct-only group (0.84 ± 0.01 gHA/cm^3^) (Fig. 4E).

## Histological and histomorphometrical analyses

**Fig. 5:**
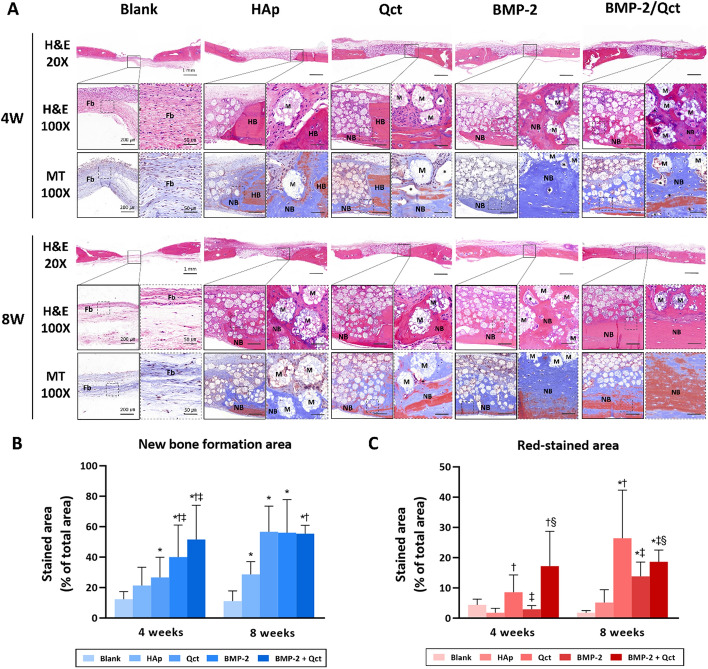
Histological sections and histomorphometrical analysis of calvarial bone defects. **A** Representative images of H&E and Masson’s trichrome stains at 4 and 8 weeks. **B **Percentage of new bone formed area in H&E stains (blue bar), and **C** red-stained area in Masson’s trichrome stains at 4 and 8 weeks. Scale bar = 1 mm (×20 images), 200 µm (×100 images), and 50 µm (×400 images in the dotted line box). Fb: fibrous tissues; NB: newly formed bone; HB: host bone; M: implanted beads; asterisk: blood vessel. *p* < 0.05 compared to the blank (*), HAp (†), Qct (‡), and BMP-2 (§) groups

**Fig. 6:**
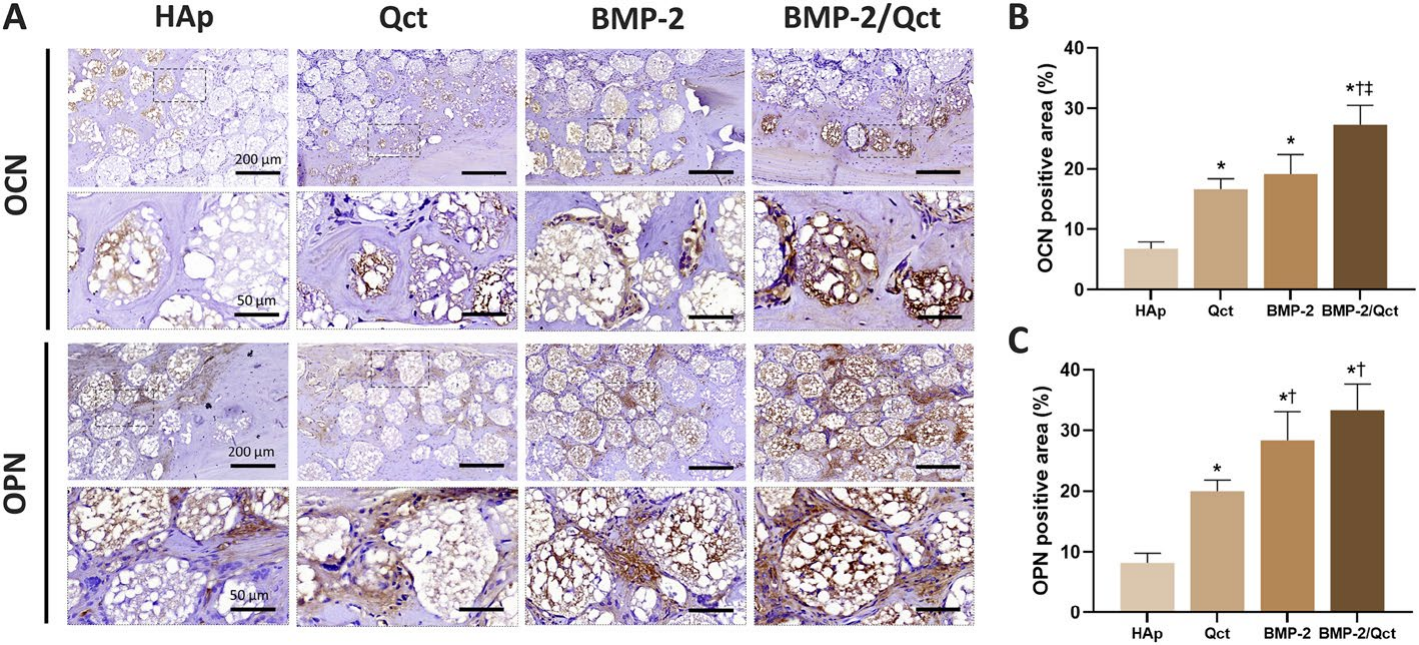
Immunohistochemical staining of calvarial bone defects at 8 weeks. **A** Immunohistochemical staining against anti-osteocalcin (OCN) and anti-osteopontin (OPN) was performed to observe osteogenesis in calvarial defects. Brown color represents positive staining. Scale bar = 200 µm (×100 images), and 50 µm (×400 images in the dotted line box). **B**, **C** OCN and OPN-positive area were calculated by ImageJ software. *p* < 0.05 compared to HAp (*), Qct (†), and BMP-2 (‡) groups

H&E and Masson’s trichrome staining were performed to evaluate the regenerated bone tissue within the defect. In general, the majority of new bones grew from the margin of the host bone. Osteoid and mineralized bone tissue grew around some of the beads in the bead-transplanted group. In contrast, in the negative control group at 4 and 8 weeks, bone formation was limited to the periphery and the central region was mostly filled with fibrous tissue (Fig. 5A). Histomorphometrical analysis revealed that the new bone formation area of both BMP-2 (39.96 ± 21.07%) and BMP-2/Qct (51.51 ± 22.57%) groups was substantially higher at 4 weeks than in the other groups, but there was no significant difference between the two groups. At 8-week post-surgery, the Qct (56.66 ± 16.81%), BMP-2 (55.99 ± 21.78%), and BMP-2/Qct groups (55.34 ± 5.53%) had a greater new bone production area than the bead control group (28.65 ± 8.35%), displaying comparable values. The red-stained area ratio of Masson’s trichrome stain, which indicates mature bone, was highest in the BMP-2/Qct group (17.19 ± 11.52%) at 4 weeks and in the Qct group (26.39 ± 15.92%) at 8 weeks, followed by the BMP-2/Qct group (18.61 ± 3.86%) (Fig. 5B and C). Immunohistochemical analysis of osteoblast biomarkers revealed that OCN-positive cells were usually present in the implanted beads surrounded by newly formed bone, and the OPN-positive area was detected both inside the beads and in the extracellular matrix of the osteoid tissue (Fig. 6A). The OCN-positive area in the BMP-2/Qct group was significantly larger than in other groups, and the OPN-positive area tended to be greater in the BMP-2/Qct and BMP-2 groups (Fig. 6B and C).

## Discussion

To function as an ideal synthetic bone graft, it is important for the osteoconductive scaffold to elicit host stem cell recruitment and osteogenesis via the release of growth factors [28]. In this study, an electrostatic spraying technique was employed to create HAp microbeads that satisfied the above criteria, and production parameters were established and optimized. Furthermore, when BMP-2 and quercetin were impregnated to assess their function as delivery carriers, bone regeneration was enhanced both in vitro and in vivo.

The manufactured beads had a microscale size and rough surface. Most beads were less than 200 μm in size and had a relatively narrow size distribution. Therefore, they can be transferred easily into the body in a minimally invasive or non-invasive manner, such via vaccine syringe [29]. Owing to the relatively narrow size distribution compared to typical methods, such as spray drying and emulsion, the manufactured beads show high productivity. In addition, the surface roughness of implants can activate the healing phenotype of macrophages as well as accelerate mineralization, osseointegration, and angiogenesis in vivo [30–32]. Based on the observation that foreign body giant cells were attached around the implanted beads, the rough surface characteristics of the ceramic beads used in this study facilitated macrophage adhesion and elongation. Following biomaterial implantation, the macrophages migrate to the tissue–implant interface, adhere to the surface, and then activate and secrete proinflammatory cytokines at the implant site, resulting in extracellular degradation of the scaffold and bone formation in the surrounding environment [33]. Consequently, these findings suggest that electrostatic spraying can produce small-sized hydroxyapatite granules uniformly and efficiently, and that the surface characteristics of the beads induce a suitable immune response in the host for bone formation.

Moreover, the smaller size and surface features of the fabricated beads may be promising for high loading efficiency and long-term delivery of phytochemical drugs and growth factors to act as functional bone substitutes [34]. In this study, BMP-2 protein and quercetin were loaded into the beads via adsorption to introduce osteogenic differentiation potential. The loading efficiency of quercetin was found to be approximately 20%, which is relatively lower than the typical drug-loading efficiency. This is due to the limited solubility of quercetin in water and the washing process with ethanol to eliminate unbound excess quercetin. However, it is possible that quercetin released at high doses can induce side effects, such as cell cytotoxicity, during cell culture in vitro and in vivo [35, 36]. In addition, when loaded into the ceramic structure, quercetin drugs can be emitted over a long-term period of over 3 months [37]. Therefore, it may not be a critical weakness for a drug delivery carrier. In contrast, the loading efficiency of BMP-2 was approximately 40%, which is a reasonable value for protein delivery [38]. Interestingly, the loaded release behavior of BMP-2 was not significantly different irrespective of quercetin loading. Quercetin, a hydrophobic phytochemical drug, had no effect on BMP-2 loading efficiency or release behavior. However, the quercetin release of the quercetin-loaded group was slightly higher than that of both the quercetin- and BMP-2-loaded groups. Because BMP-2 was loaded sequentially after quercetin loading, a part of loaded quercetin might have leaked away during the incubation and washing steps during BMP-2 introduction. Although the initial quercetin release in the quercetin-loaded group was slightly lower, the release trend remained the same throughout the entire test period. Thus, it was confirmed that hydroxyapatite beads can serve as a delivery vehicle that enables the sustained release of osteogenic drugs. We observed that loaded BMP-2 and quercetin had a synergistic effect on inducing osteogenesis of the cells in vitro. However, the BMP-2 still showed initial burst release behavior from the microbead. In addition, quercetin showed relatively slow release behavior in aqueous media due to its low water solubility. Slow and sustained release behavior is an attractive property in drug delivery systems. However, it might be ineffective due to the lower level of release drug under a therapeutic window. We need to consider improving the stable protein and drug-releasing properties of the microbead.

Next, we investigated the safety and bioactivity of the quercetin and BMP-2 released from the beads. The administered quantity of BMP-2 was 1 μg per defect, which is a low dose. However, previous studies using a 5-mm calvarial defect [39–41] showed a considerable increase in bone production with 1 μg BMP-2, and our data showed that the bone volume of the BMP-2 group was 2.5 times greater than that of the empty defect. With regard to the amount of quercetin, certain studies have reported the dose-dependent cytotoxicity of quercetin to cancer cell lines [35, 42]. However, the in vitro DNA content of osteoblast-like cells after co-culturing with a quercetin-loaded implant was comparable to that of other groups that did not include quercetin. In addition, when each bead was transplanted into the subcutaneous tissue and calvarial bone defects, a tissue irritation response, such as redness or edema, was not observed in any animal, and foreign body giant cell count results (Additional file 1: Fig. S1) showed no significant difference between the drug-loaded and control groups at 4-week post-implantation. Therefore, the doses of quercetin and BMP-2 released from the implant in this study are considered physiologically safe, based on the in vitro and in vivo results. Although it is known that HAp granules take many years to degrade under physiological conditions [43], the degradation of fabricated implants has a microscale size and is expected to occur through the cell-mediated resorption process [7, 44]. Histological sections at 8-week post-surgery showed multinucleated giant cells around the implants, and macrophages intruded into the majority of the implanted beads, indicating that they are highly biodegradable. However, further studies on the long-term degradation are required. Our findings indicate that the amounts of BMP-2 and quercetin released from the beads are safe and sufficient to be biologically active.

The osteogenic efficiency of quercetin and/or BMP-2-loaded beads was evaluated in vitro and in vivo. BMP-2 protein is a well-known osteogenic inducible growth factor [45]. In these quantification data in the BMP-2-loaded group, the quantitative results of DNA and ALP were higher than in the other groups, indicating that loaded BMP-2 could significantly induce the proliferation and osteogenic differentiation of the cells. Unfortunately, these properties of quercetin-loaded groups were not meaningful compared to those of the non-loaded group, and the values of the BMP-2/Qct group were also not remarkable compared to those of the BMP-2 and quercetin-loaded groups. However, gene expression trends of the cultured cells were quite different in comparison with quantitative analysis using the assay kit. Our results showed that ALP and RUNX2 gene expressions in the Qct group were significantly higher than those in the non-loaded group, which is consistent with previous research showing that quercetin promotes BMSC proliferation and mRNA expression of RUNX2, BMP-2, and OCN genes, and improving BMSC bone differentiation [46]. ALP is an early osteogenic marker that plays an important role in maturation. RUNX2 is an important transcription factor associated with osteogenic differentiation and is regulated by the Wnt signaling pathway for bone repair and regeneration [47, 48]. The expression of these genes was the highest in the BMP-2/Qct group, indicating that BMP-2 and quercetin could synergistically affect osteogenic cells in the induction of osteogenic differentiation. The Alizarin Red S staining images represent the results of the biological assay and gene expression analysis. The positive signals in the BMP-2/Qct-loaded group were appreciably stronger than those in the other groups, which was shown to have increased calcium formation. This indicates that the BMP-2-and-quercetin-loaded beads have a promising potential to induce osteogenic differentiation as functional bone substitutes in vitro.

To date, the comparative efficacy of BMP-2 and quercetin, as well as the synergistic effect of both medications on in vivo bone regeneration, has not been explored. Using micro-CT data to compare the bone regeneration efficacy of BMP-2 and quercetin, the BMP-2 group had greater bone volume at both 4 and 8-week post-operation than the Qct group. As 90% of BMP-2 is released in the first week, it is assumed that the high level of osteogenesis at 4 weeks after implantation is a result of the promotion of early cell migration and proliferation. The new bone volume in the BMP-2 group did not change significantly between weeks 4 and 8. However, in the case of quercetin, a higher bone volume was measured than in the 4th week, which might be interpreted as evidence of the sustained release of quercetin. In addition, the BMP-2/Qct group in the 8th week showed a substantial increase in bone volume compared to the BMP-2 group. Because the addition of quercetin did not change the emission pattern of BMP-2, this can be thought of as a synergistic effect owing to the initial bone-regeneration effect of BMP-2 and the long-term release of quercetin. In addition, the results of the histomorphological analysis of the Masson trichrome staining indicate that the red-stained area, which indicates mineralized bone, is significantly higher in the BMP-2/Qct group than in the BMP-2 group, suggesting the osteoregulatory effect of quercetin, which inhibits osteoclastogenesis and stimulates osteogenesis. Therefore, our findings imply that combining quercetin with BMP-2 has a synergistic effect on promoting bone mineralization in an in vivo bone defect model, in accordance with the in vitro results.

## Conclusions

In this study, HAp microparticles were manufactured with a uniform size of 50–150 μm using electrostatic spraying without complicated conditions or procedural steps. It was also confirmed that the novel HAp microbeads could act as osteoconductive carriers of BMP-2 and quercetin through in vitro and in vivo experiments. In addition, it was proven that only quercetin-loaded beads increased bone formation when compared to the control group, and the simultaneous application of both BMP-2 and quercetin via HAp beads had a synergistic effect on bone regeneration in rat calvarial defect model. Therefore, it was demonstrated that electrostatic spraying can prove to be an efficient way to fabricate ceramic granules, and HAp microbeads containing both BMP-2 and quercetin can be applied as effective implants for bone defect treatment.

## Materials and methods

### Preparation and characterization of HAp beads

Sodium alginate (Pronova UP MVG; FMC Biopolymer AS, Sandvika, Norway) was dissolved in distilled water at a concentration of 1 wt% (w/v) and 6–8 wt% (w/v) HAp (particle size = 50 nm, max., CGBio Inc., Seongnam, Korea) was mixed with a homogenous dissolved alginate solution using a planetary centrifugal mixer and a three-dimensional ultrasonic mixer for 6 min and 15–30 min, respectively. Microsized spherical hybrid structures were fabricated with a mixture of alginate and HAp using an encapsulator (B-390; Buchi, Flawil, Switzerland) in the frequency range of 1500–4000 Hz, electrode range of 1000–2000 V, and pressure range of 150–400 mbar. The fabricated structures were crosslinked in 100 mM CaCl_2_ (Sigma, St. Louis, MO, USA) for 30 min under stirring. Crosslinked structures were obtained from the CaCl_2_ solution and washed with ethanol. After drying overnight at room temperature, the samples were sintered at 1200 °C. The phase composition of the sintered microbeads was determined via X-ray diffraction (XRD, DMAX-2500; Rigaku, Tokyo, Japan) at an operating voltage of 40 kV. Measurements were taken in the 2θ angle range of 5–55°. Images of the sintered beads were obtained using a tabletop SEM (SNE-4500 M Plus; SEC Co., Ltd., Suwon, Korea).

### In vitro release profiles of BMP-2 and quercetin from HAp beads

To load quercetin on the surface of the microbeads via adsorption, 8 wt% (w/w) quercetin hydrate (TCI, Tokyo, Japan) was dissolved in ethanol and placed into a glass vial containing sintered microbeads. After overnight incubation with rotation, both the non-loaded and quercetin-loaded microbeads were washed with ethanol to remove excess quercetin and dried at room temperature overnight. Each group was loaded with 5.0 μg human recombinant BMP-2 (Peprotech, Rocky Hill, NJ, USA) and incubated for 6 h at 37 °C in a cell culture incubator. The BMP-2-loaded microbeads were centrifuged at 15,800*g* for 5 min and the supernatant was aspirated. After centrifugation at 15,800*g* for 5 min, the supernatant was aspirated again and immersed in PBS. At each timepoint, the microbeads were centrifuged at 15,800*g* for 5 min, and the obtained supernatant was used to measure the release behavior of quercetin and BMP-2, which were investigated using a spectrophotometer at 375 nm and an ELISA kit (Peprotech), respectively. Fresh PBS was added to each microbead group for continuous release into the buffer.

### Osteogenic differentiation and cytotoxicity of MG-63 co-cultured with HAp beads

5 × 10^4^ cells of the human osteosarcoma cell line MG-63 were seeded into each well of a 24-well cell culture plate and incubated in a cell culture incubator at 37 °C under a 5% CO_2_ atmosphere. After 6 h of incubation, the medium was removed, and the cells were washed with PBS to remove unbound cells. The BMP-2-and/or quercetin-loaded HAp microbeads were immersed in osteogenic differentiation media containing 10% FBS, 1% penicillin/streptomycin, 50 μg/ml ascorbic acid (TCI), 100 nM dexamethasone (Sigma), and β-glycerophosphate (Sigma), and transferred into a cell-seeded well plate with a polyethylene terephthalate trans-membrane insert (SPLInsert™ Hanging;SPL Life Sciences, Pocheon, Korea). Fresh osteogenic differentiation medium was supplied every alternate day for 2 weeks. To quantify alkaline phosphatase (ALP) activity and DNA content, cells were washed twice with PBS and treated with cell lysis buffer (CelLytic™ M; Sigma). After incubation for 5 min, the retrieved solutions were transferred into each tube and centrifuged at 12,000 rpm for 5 min. ALP activity was measured using *p*-nitrophenyl phosphate (pNPP) as an alkaline phosphatase substrate, using a spectrophotometer at a wavelength of 405 nm. The DNA content was measured using the Quant-iT™ dsDNA Assay Kit (Invitrogen, Carlsbad, CA, USA). To evaluate ALP and RUNX2 gene expression as osteogenic marker genes, cultured cells in osteogenic differentiation media were treated with RNA extraction reagent (RNAiso PLUS; Takara, Shiga, Japan). cDNA was synthesized using Maxime™ RT Premix (iNtRON Biotechnology, Seongnam, Korea), and quantitative gene expression was detected through real-time PCR equipment (TP900; Takara) using real-time PCR master mix (GoTaq®; Promega, Madison, WI, USA). The following primer sequences were used (Integrated DNA Technologies, Coralville, IA, USA): ALP, 5′-ACG TGG CTA AGA ATG TCA TC-3′ and 5′-CTG GTA GGC GAT GTC CTT A-3′; RUNx2, 5′-CCA GAT GGG ACT GTG GTT ACT G-3′ and 5′-CGG AGC TCA GCA GAA TAA TTT TC-3′; GAPDH, 5′-CCC TCC AAA ATC AAG TGG GG-3′ and 5′-CGC CAC AGT TTC CCG GAG GG-3′. To estimate calcium formation in the cells, cultured cells were washed once with PBS and fixed in a 4% formaldehyde solution for 1 h. After removing the solution, the cells were again washed with PBS and stained with 2% Alizarin Red S (pH 4.2) for 2 min. Stained cells were continuously washed with deionized water until no excess dye was present. Images of stained cells were obtained using an optical microscope (IX71; Olympus, Tokyo, Japan).

### In vivo bone regeneration in a rat calvarial defect model

Thirty-two 16-week-old male Sprague–Dawley rats, weighing 400–500 g, were used to assess in vivo bone regeneration. Rats were allowed to acclimate to their diet, water, and housing for 1 week prior to surgery. Before the surgical procedure was carried out, rats were anesthetized with 4 MAC of isoflurane (Hana Pharm, Seoul, Korea) in the induction cage and maintained with 1.5 ~ 2 MAC via a face mask at an O_2_ flow rate of 1–2 L/min. Tramadol (12.5 mg/kg; Hanall Biopharma, Seoul, Korea) and enrofloxacin (Baytril®, 5 mg/kg; Bayer, Leverkusen, Germany) were injected subcutaneously as preoperative analgesic and antibiotic, respectively. The surgical site was clipped, disinfected with chlorhexidine–alcohol solution, and draped with a sterile drape. The skin was incised on the midline of the skull, and the subcutaneous tissue and periosteum were incised and retracted to expose the calvarium. Bilateral calvarial defects, 5 mm in diameter, were generated using a trephine burr with a dental unit and flushed with normal saline to avoid overheating. Each bone defect was filled with 20 mg ceramic beads. Only ceramic beads were implanted in the bead control group, BMP-2 (1 µg/defect) or quercetin (8 wt%)-loaded ceramic beads were implanted in the experimental groups, and the defects were left empty in the negative control group. Fibrin glue (Greenplast®; Green Cross, Seoul, Korea) was applied to the implanted beads to prevent their migration. The periosteum, muscle, and subcutaneous tissue were sutured using 4–0 absorbable suture material (Ethicon, Edinburgh, UK), and the skin was closed using a 4–0 nylon monofilament suture (AILEE Co., Busan, Korea). All animal experimental procedures were approved by the Institutional Animal Care and Use Committee of the Seoul National University (SNU-200612-4).

### Micro-computed tomography (micro-CT) analysis

The rats were euthanized via CO_2_ inhalation at 4- and 8-week post-implantation. The calvarium, which includes the parietal bones as well as parts of the frontal and occipital bones, was obtained. The harvested calvaria were immediately fixed in 10% neutral-buffered formalin at room temperature. After 2 days, the samples were transferred to PBS and kept at 4 °C until micro-CT examination. All the specimens were scanned using a Skyscan 1272 micro-CT scanner (Bruker, Kontich, Belgium). Scanning was performed using the following parameters: 80 kV voltage, 125 μA current, and a 1.0-mm aluminum filter. After standardized reconstruction using the NRecon software, the acquired data were analyzed using CTAn (Bruker micro-CT) and ImageJ software. Bone growth was quantified by selecting a cylindrical volume of interest in the axial plane above the defect sites with a 5-mm diameter and 1-mm height. Outcome measures included bone volume (BV), bone volume fraction (BV/TV), and bone mineral density (BMD).

### Histological and histomorphometrical analysis

Following sample harvest or micro-CT analysis, the fixed specimens were decalcified in 10% ethylenediaminetetraacetic acid solution (pH 7.4) at room temperature and processed for paraffin embedding. Paraffin blocks were sectioned at a thickness of 5 µm using a microtome. The calvarial sections were stained with hematoxylin and eosin (H&E) and Masson’s trichrome to examine the newly formed tissues. ImageJ was utilized to measure and analyze the new bone formed area in H&E stains and the red-stained area in Masson’s trichrome stains of the calvarial tissue sections. For immunohistochemistry, the calvarial sections were stained with anti-OCN (Santa Cruz Biotechnology Inc., Santa Cruz, CA, USA) and anti-OPN antibodies (Abcam, Cambridge, UK). Primary antibodies against anti-OCN (Cat# sc-365797, 1:200) and anti-OPN (Cat# ab63856, 1:200) were incubated at 4 °C overnight. The HRP-conjugated secondary antibodies (Vector Laboratories, Inc., Burlingame, CA, USA) against the primary antibody were applied for 1 h at room temperature, and the sections were incubated for 10 min with the diaminobenzidine substrate (ImmPACT® DAB; Vector Laboratories, Inc.) to detect signals. The OCN and OPN-positive area were evaluated by ImageJ (NIH) using the Colour Deconvolution 2 plugin.

### Statistical analysis

All quantitative data were expressed as mean ± standard deviation (SD). Statistical analysis was performed using GraphPad Prism (GraphPad Software, version 8.0.1). Statistical significance between groups was determined using the Kruskal–Wallis and Mann–Whitney tests. Values with *p* values less than 0.05 (*p* < 0.05) were considered significant.

## Supplementary Information

**Additional file 1. **In vivo subcutaneous transplantation results.

## Data Availability

The data sets used and/or analysed during the current study are available from the corresponding author on reasonable request.

## Abbreviations

ALP: Alkaline phosphatase
BMD: Bone mineral density
BMP-2: Bone morphogenetic protein-2
BV: Bone volume
BV/TV: Bone volume fraction
HAp: Hydroxyapatite
H&E: Hematoxylin and eosin
Micro-CT: Micro-computed tomography
OCN: Osteocalcin
OPN: Osteopontin
pNPP: *p*-Nitrophenyl phosphate
Qct: Quercetin
SD: Standard deviation

## References

[1] Arrington ED, Smith WJ, Chambers HG, Bucknell AL, Davino NA (1996). Complications of iliac crest bone graft harvesting. Clin Orthopaedics Related Res..

[2] Betz RR (2002). Limitations of autograft and allograft: new synthetic solutions. Orthopedics.

[3] Bohner M, Galea L, Doebelin N (2012). Calcium phosphate bone graft substitutes: failures and hopes. J Eur Ceram Soc.

[4] Kumar Y, Nalini K, Menon J, Patro DK, Banerji B (2013). Calcium sulfate as bone graft substitute in the treatment of osseous bone defects, a prospective study. J Clin Diagn Res.

[5] Granel H, Bossard C, Nucke L, Wauquier F, Rochefort GY, Guicheux J (2019). Optimized bioactive glass: the quest for the bony graft. Adv Healthc Mater.

[6] Saito N, Takaoka K (2003). New synthetic biodegradable polymers as BMP carriers for bone tissue engineering. Biomaterials.

[7] Jeong J, Kim JH, Shim JH, Hwang NS, Heo CY (2019). Bioactive calcium phosphate materials and applications in bone regeneration. Biomater Res.

[8] Albrektsson T, Johansson C (2001). Osteoinduction, osteoconduction and osseointegration. Eur Spine J.

[9] Yoshikawa H, Myoui A (2005). Bone tissue engineering with porous hydroxyapatite ceramics. J Artif Organs.

[10] Patel N, Best S, Bonfield W, Gibson IR, Hing K, Damien E (2002). A comparative study on the in vivo behavior of hydroxyapatite and silicon substituted hydroxyapatite granules. J Mater Sci - Mater Med.

[11] Wang G, Qiu J, Zheng L, Ren N, Li J, Liu H (2014). Sustained delivery of BMP-2 enhanced osteoblastic differentiation of BMSCs based on surface hydroxyapatite nanostructure in chitosan–HAp scaffold. J Biomater Sci Polym Ed.

[12] Sun R, Lu Y, Chen K (2009). Preparation and characterization of hollow hydroxyapatite microspheres by spray drying method. Mater Sci Eng, C.

[13] Chen B-H, Chen K-I, Ho M-L, Chen H-N, Chen W-C, Wang C-K (2009). Synthesis of calcium phosphates and porous hydroxyapatite beads prepared by emulsion method. Mater Chem Phys.

[14] Ameri M, Maa Y-F (2006). Spray drying of biopharmaceuticals: stability and process considerations. Drying Technol.

[15] Jenjob R, Phakkeeree T, Seidi F, Theerasilp M, Crespy D (2019). Emulsion techniques for the production of pharmacological nanoparticles. Macromol Biosci.

[16] Athanasiou VT, Papachristou DJ, Panagopoulos A, Saridis A, Scopa CD, Megas P (2009). Histological comparison of autograft, allograft-DBM, xenograft, and synthetic grafts in a trabecular bone defect: an experimental study in rabbits. Med Sci Monit.

[17] Noshi T, Yoshikawa T, Dohi Y, Ikeuchi M, Horiuchi K, Ichijima K (2001). Recombinant human bone morphogenetic protein-2 potentiates the in vivo osteogenic ability of marrow/hydroxyapatite composites. Artif Organs.

[18] Rengachary SS (2002). Bone morphogenetic proteins: basic concepts. Neurosurg Focus.

[19] Oryan A, Alidadi S, Moshiri A, Bigham-Sadegh A (2014). Bone morphogenetic proteins: a powerful osteoinductive compound with non-negligible side effects and limitations. BioFactors.

[20] Kempen DH, Lu L, Heijink A, Hefferan TE, Creemers LB, Maran A (2009). Effect of local sequential VEGF and BMP-2 delivery on ectopic and orthotopic bone regeneration. Biomaterials.

[21] Kwak EJ, Cha IH, Nam W, Yook J, Park YB, Kim H (2018). Effects of locally administered rh BMP-2 and bisphosphonate on bone regeneration in the rat fibula. Oral Dis.

[22] Li L, Zhou G, Wang Y, Yang G, Ding S, Zhou S (2015). Controlled dual delivery of BMP-2 and dexamethasone by nanoparticle-embedded electrospun nanofibers for the efficient repair of critical-sized rat calvarial defect. Biomaterials.

[23] Rauf A, Imran M, Khan IA, Ur-Rehman M, Gilani SA, Mehmood Z (2018). Anticancer potential of quercetin: a comprehensive review. Phytother Res.

[24] Dajas F (2012). Life or death: neuroprotective and anticancer effects of quercetin. J Ethnopharmacol.

[25] Lesjak M, Beara I, Simin N, Pintać D, Majkić T, Bekvalac K (2018). Antioxidant and anti-inflammatory activities of quercetin and its derivatives. J Funct Foods.

[26] Wong RW, Rabie ABM (2008). Effect of quercetin on bone formation. J Orthop Res.

[27] Forte L, Torricelli P, Boanini E, Gazzano M, Rubini K, Fini M (2016). Antioxidant and bone repair properties of quercetin-functionalized hydroxyapatite: an in vitro osteoblast–osteoclast–endothelial cell co-culture study. Acta Biomater.

[28] Haugen HJ, Lyngstadaas SP, Rossi F, Perale G (2019). Bone grafts: which is the ideal biomaterial?. J Clin Periodontol.

[29] Zhong H, Chan G, Hu Y, Hu H, Ouyang D (2018). A comprehensive map of FDA-approved pharmaceutical products. Pharmaceutics.

[30] Chehroudi B, Ghrebi S, Murakami H, Waterfield JD, Owen G, Brunette DM (2010). Bone formation on rough, but not polished, subcutaneously implanted Ti surfaces is preceded by macrophage accumulation. J Biomed Mater Res Part A.

[31] Wennerberg A, Albrektsson T, Andersson B (1995). An animal study of cp titanium screws with different surface topographies. J Mater Sci Mater Med.

[32] Madden LR, Mortisen DJ, Sussman EM, Dupras SK, Fugate JA, Cuy JL (2010). Proangiogenic scaffolds as functional templates for cardiac tissue engineering. Proc Natl Acad Sci.

[33] Barbeck M, Booms P, Unger R, Hoffmann V, Sader R, Kirkpatrick CJ (2017). Multinucleated giant cells in the implant bed of bone substitutes are foreign body giant cells—new insights into the material-mediated healing process. J Biomed Mater Res, Part A.

[34] Ortega-Oller I, Padial-Molina M, Galindo-Moreno P, O’Valle F, Jódar-Reyes AB, Peula-García JM (2015). Bone regeneration from PLGA micro-nanoparticles. BioMed Res Int.

[35] Srivastava S, Somasagara RR, Hegde M, Nishana M, Tadi SK, Srivastava M (2016). Quercetin, a natural flavonoid interacts with DNA, arrests cell cycle and causes tumor regression by activating mitochondrial pathway of apoptosis. Sci Rep.

[36] Mohammed HA, Sulaiman GM, Anwar SS, Tawfeeq AT, Khan RA, Mohammed SA (2021). Quercetin against MCF7 and CAL51 breast cancer cell lines: apoptosis, gene expression and cytotoxicity of nano-quercetin. Nanomedicine.

[37] Raja N, Park H, Choi Y-J, Yun H-S (2021). Multifunctional calcium-deficient hydroxyl apatite-alginate core–shell-structured bone substitutes as cell and drug delivery vehicles for bone tissue regeneration. ACS Biomater Sci Eng.

[38] Zhou P, Wu J, Xia Y, Yuan Y, Zhang H, Xu S (2018). Loading BMP-2 on nanostructured hydroxyapatite microspheres for rapid bone regeneration. Int J Nanomed.

[39] Chung Y-I, Ahn K-M, Jeon S-H, Lee S-Y, Lee J-H, Tae G (2007). Enhanced bone regeneration with BMP-2 loaded functional nanoparticle–hydrogel complex. J Control Release.

[40] Rahman CV, Ben-David D, Dhillon A, Kuhn G, Gould TW, Müller R (2014). Controlled release of BMP-2 from a sintered polymer scaffold enhances bone repair in a mouse calvarial defect model. J Tissue Eng Regen Med.

[41] Ben-David D, Srouji S, Shapira-Schweitzer K, Kossover O, Ivanir E, Kuhn G (2013). Low dose BMP-2 treatment for bone repair using a PEGylated fibrinogen hydrogel matrix. Biomaterials.

[42] Li N, Sun C, Zhou B, Xing H, Ma D, Chen G (2014). Low concentration of quercetin antagonizes the cytotoxic effects of anti-neoplastic drugs in ovarian cancer. PLoS ONE.

[43] Oonishi H, Hench L, Wilson J, Sugihara F, Tsuji E, Kushitani S (1999). Comparative bone growth behavior in granules of bioceramic materials of various sizes. J Biomed Mater Res.

[44] Habraken W, Habibovic P, Epple M, Bohner M (2016). Calcium phosphates in biomedical applications: materials for the future?. Mater Today.

[45] Bessa PC, Casal M, Reis R (2008). Bone morphogenetic proteins in tissue engineering: the road from laboratory to clinic, part II (BMP delivery). J Tissue Eng Regen Med.

[46] Pang X-G, Cong Y, Bao N-R, Li Y-G, Zhao J-N (2018). Quercetin stimulates bone marrow mesenchymal stem cell differentiation through an estrogen receptor-mediated pathway. BioMed Res Int.

[47] Thiagarajan L, Abu-Awwad HA-DM, Dixon JE (2017). Osteogenic programming of human mesenchymal stem cells with highly efficient intracellular delivery of RUNX2. Stem Cells Transl Med.

[48] Shui C, Spelsberg TC, Riggs BL, Khosla S (2003). Changes in Runx2/Cbfa1 expression and activity during osteoblastic differentiation of human bone marrow stromal cells. J Bone Miner Res.

