# Supplementary material for: Hydroxyapatite microbeads containing BMP-2 and quercetin fabricated via electrostatic spraying to encourage bone regeneration
Source: Biomed Eng Online. 2023 Feb 18;22:15. doi: 10.1186/s12938-023-01078-y (PMC9938985; doi:10.1186/s12938-023-01078-y)
Supplement: Supplementary file 1 — Additional file 1. In vivo subcutaneous transplantation results. [file 12938_2023_1078_MOESM1_ESM.docx]

Supplementary Information

**Hydroxyapatite microbeads containing BMP-2 and quercetin
fabricated via electrostatic spraying to encourage bone regeneration**

Seoyun Lee^1,2,†^, Honghyun Park^3,†^, Jeong-Seop Oh^4^, Kyubin Byun^3,5^, Dae-Yong Kim^4^, Hui-suk Yun^3,5,*^, Byung-Jae Kang^1,2,*^

^1^ Department of Veterinary Clinical Sciences, College of Veterinary Medicine and Research Institute for Veterinary Science, Seoul National University, Seoul 08826, South Korea

^2^ BK21 FOUR Future Veterinary Medicine Leading Education and Research Center, Seoul National University, Seoul 08826, South Korea

^3^ Department of Advanced Biomaterials Research, Ceramics Materials Division, Korea Institute of Materials Science (KIMS), Changwon 51508, South Korea

^4^ Department of Veterinary Pathology, College of Veterinary Medicine, Seoul National University, Seoul 08826, South Korea

^5^ Department of Advanced Materials Engineering, University of Science & Technology (UST), Daejeon 34113, South Korea

^†^ Seoyun Lee and Honghyun Park contributed equally to this work.

* Correspondence: bjkang81@snu.ac.kr; yuni@kims.re.kr

**Fig. S1** *in vivo* subcutaneous transplantation of hydroxyapatite microbeads loaded with BMP-2 or quercetin. A total of 20 mg of each bead type was subcutaneously transplanted to the rat’s dorsal region: HAp, Low Qct (1 wt%), High Qct (8 wt%), BMP-2 (1 ug/20 mg beads), Low Qct+BMP-2, and High Qct+BMP-2 beads. **A** The representative photomicrograph images of H&E stains with the bead-transplanted area at 1 and 4 weeks post implantation. Scale bar = 1 mm. Foreign body giant cell was counted per 1 mm^2^ within the bead-containing area 1 week (**B**) and 4 weeks (**C**) after surgery. p < 0.05 compared to HAp (*) and Low Qct (†) groups.


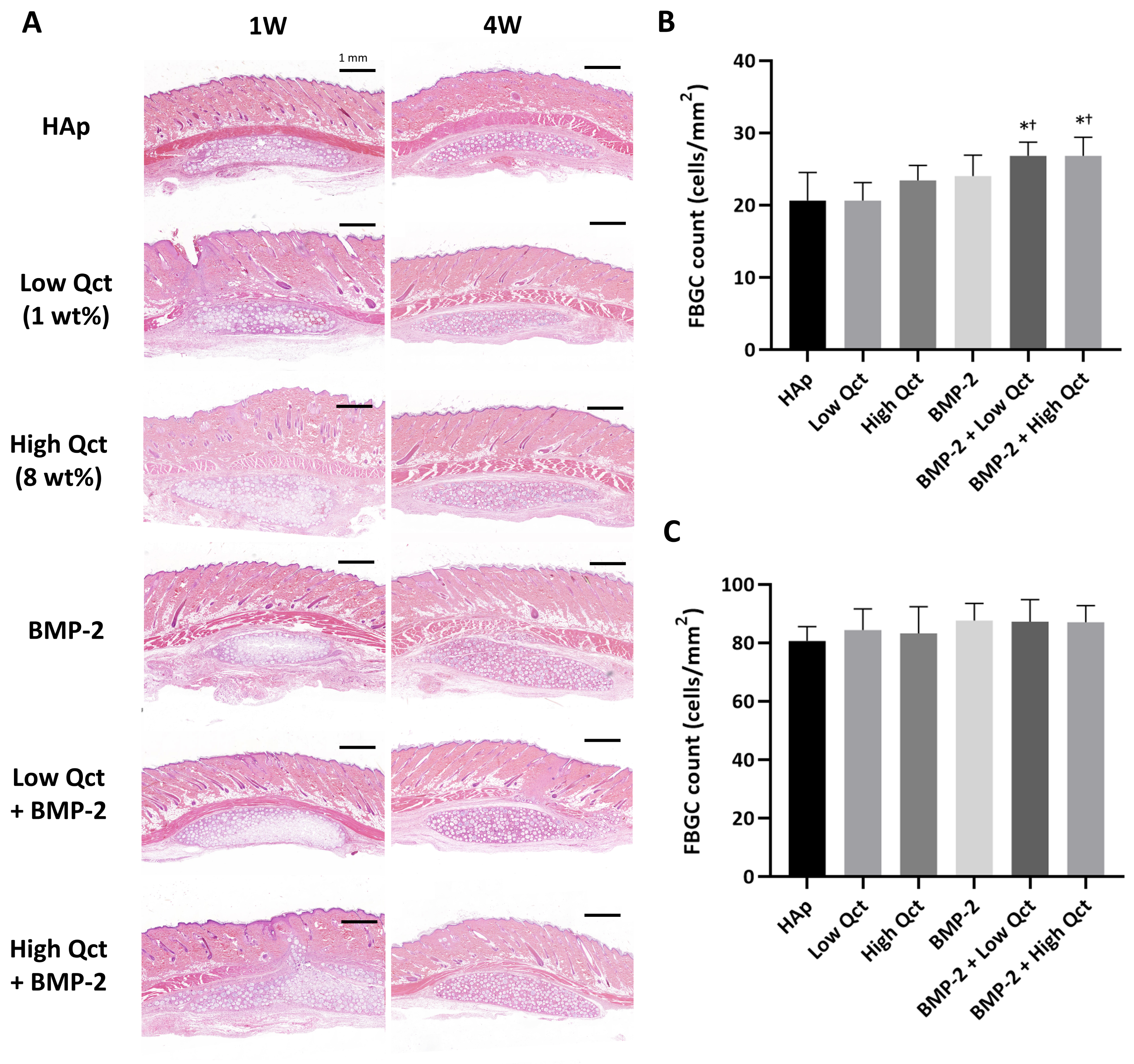


**Materials and Methods**

To evaluate the biocompatibility of HAp microbeads containing BMP-2 and quercetin, 20 mg of each bead type was subcutaneously implanted into the dorsum of rats. Twelve 6-week-old male Sprague-Dawley rats, weighing 200–300 g, were used. Twelve rats were randomly assigned to 1- or 4-week groups, and 6 different types of implants were transplanted to each rat’s dorsal region: HAp, Low Qct (1 wt%), High Qct (8 wt%), BMP-2 (1 ug/20 mg beads), Low Qct+BMP-2, and High Qct+BMP-2 beads. Following a week of acclimation, the rats underwent anesthesia and skin preparation for surgery, as described above. After the skin was incised, a subcutaneous pouch was made, and different types of beads were transplanted into the pouch. Each implant site was rotated clockwise to eliminate location bias. After implantation, the subcutaneous tissue and skin were sutured using 4-0 Vicryl (Ethicon) and 4-0 nylon suture (AILEE Co.), respectively. All surgical sites were disinfected daily with 0.2% chlorhexidine solution for a week. After 1 and 4 weeks, the subcutaneous tissue sections were decalcified, embedded in paraffin, sectioned at 5 mm, and stained with hematoxylin and eosin (H&E) to assess the acute or chronic inflammation of the biomaterials. For histomorphometrical analysis, a cross-sectional area of 1 mm^2^ was randomly selected in the subcutaneously implanted region and multinucleated cells were manually counted.

**Results**

*In vivo* subcutaneous transplantation was performed to assess the biocompatibility of the biomaterial as well as the acute inflammatory and foreign body responses against hydroxyapatite bead implants containing BMP-2 and quercetin. The difference in the inflammatory response between the drug-loaded groups 1 week after implantation was significant in the BMP-2-containing group. In the 4-week samples, no significant differences were observed between the drug-containing groups.
